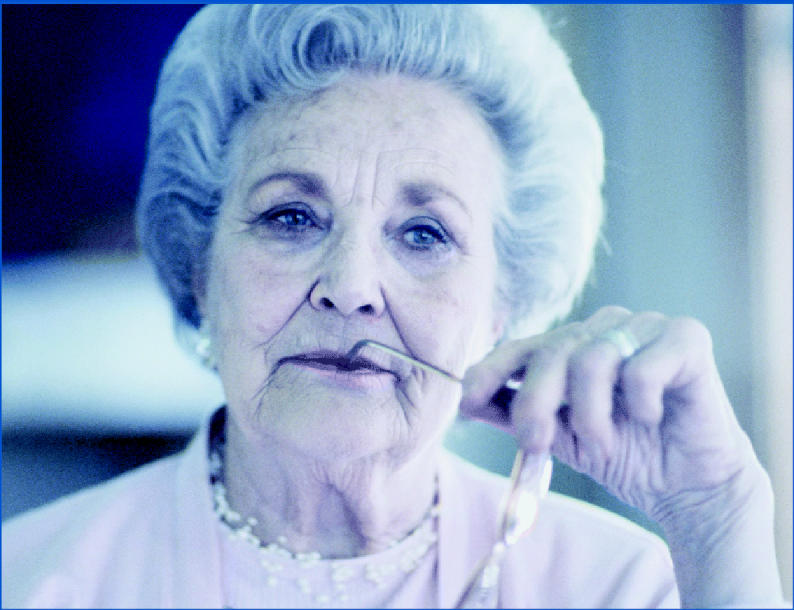# Headliners: Neurological Disease: Neural Protein May Stop the Progression of Alzheimer Disease

**Published:** 2005-01

**Authors:** Jerry Phelps

Stein TD, Anders NJ, DeCarli C, Chan SL, Mattson MP, Johnson JA. 2004. Neutralization of transthyretin reverses the neuroprotective effects of secreted amyloid precursor protein (APP) in APPSW mice resulting in tau phosphorylation and loss of hippocampal neurons: support for the amyloid hypothesis. J Neurosci 24:7707–7717.

As many as 4.5 million Americans suffer from Alzheimer disease (AD), which usually begins after age 60, and the risk of developing the disease goes up with age. About 5% of men and women aged 65–74 have AD, and nearly half of those aged 85 and older have the disease.

AD is characterized by the presence of protein plaques and tangles of fibers in brain tissue. The disease may in fact be caused by the abnormal processing of the so-called amyloid precursor protein and the accumulation of the protein β-amyloid. Other brain abnormalities in people with AD include nerve cell death in specific areas that are vital to memory and other mental abilities, as well as lower levels of certain neurotransmitters. A recent study by NIEHS grantee Jeffrey Johnson of the University of Wisconsin–Madison has identified a protein known as transthyretin that blocks the effects of β-amyloid.

In working with a transgenic mouse containing defective human genes associated with early-onset AD, Johnson and colleagues noticed that although these mice had high levels of β-amyloid, they did not exhibit any neurodegenerative symptoms. Further investigations led the team to discover that these mice also were producing high levels of transthyretin. When the mice were given antibodies that prevented transthyretin from interacting with the β-amyloid protein, the mice showed typical brain cell death. *In vitro* studies of human brain cells treated with transthyretin and β-amyloid showed minimal amounts of cell death, confirming the results seen in the mice.

These studies show that transthyretin may block the progression of AD by inhibiting the effects of β-amyloid. This discovery suggests that it may be possible to develop a drug that increases the production of transthyretin and thus protects people at risk for AD, such as those with a genetic predisposition. The findings may also improve the chances of detecting potential environmental factors in the development of AD by allowing scientists to identify agents that upset the balance between transthyretin and β-amyloid proteins.

## Figures and Tables

**Figure:**